# Reentry to the Mediastinum When the Ascending Aorta Is Adherent to the Sternum: A Two-Stage Sternotomy Approach

**DOI:** 10.21470/1678-9741-2023-0310

**Published:** 2025-02-14

**Authors:** Saimir Kuci, Ermal Likaj, Alfred Ibrahimi, Marsela Goga, Romina Teliti, Jacob Zeitani

**Affiliations:** 1 Cardiac Anesthesia and Intensive Care Unit, University Hospital Center 'Mother Teresa', Tirana, Albania.; 2 Cardiac Surgery Unit, University Hospital Center 'Mother Teresa', Tirana, Albania.; 3 Cardiology Department, Università Cattolica Nostra Signora Del Buon Consiglio, Tirana, Albania.; 4 Neuroscience and Rehabilitation Department, University of Ferrara, Ferrara, Italy.

**Keywords:** Ascending Aorta Aneurysm, Cardiopulmonary Bypass, Hypthermia, Mediastinum, Sternotomy, Sternum, Time

## Abstract

Reentry to the mediastinum when the ascending aorta aneurysm is adherent to the sternum is characterized by high risk of aneurysm rupture during sternum opening. In such cases, often cardiopulmonary bypass via peripheral vessels is instituted, and reentry done in deep hypothermia and circulatory arrest. To reduce both risks of aneurysm rupture during resternotomy and those related to prolonged cardiopulmonary bypass time, we present a surgical approach consisting of a two-stage sternotomy to avoid the risky zone and extra-anatomic epiaortic vessels anastomoses.

## INTRODUCTION

Reentry to the mediastinum, especially when the heart and/or ascending aorta are in direct contact with the sternum, is challenging, and preoperative surgical strategy plan is needed to avoid rupture and uncontrollable bleeding. The need for repeated surgery after surgical repair of acute type A aortic dissection is common, especially when only the ascending aorta has been replaced. To reduce such risks, on-pump cardiopulmonary bypass (CPB), deep cooling, and circulatory arrest prior to resternotomy have been proposed^[1,2]^. However, impaired hemostasis and coagulation abnormalities are known to be correlated to prolonged CPB time and were found to be independent risk factors for mortality^[3,4]^. Moreover, dissection of the adhesions while the patient is heparinized is more complex and time-consuming because of excessive bleeding interfering with surgeon view and apparatus efficiency.

This report describes a surgical technique consisting of a two-stage sternotomy, extra-anatomic anastomoses of the epiaortic vessels and thoracic endovascular aortic repair (TEVAR) implantation. This is designed to treat patients who had previously undergone ascending aorta replacement requiring a second operation to treat the arch and descending aorta and presenting with an aortic aneurysm adherent to the sternum.

## TECHNIQUE

Under general anesthesia and invasive blood pressure monitoring — including radial, femoral, and external temporal arteries — the left common carotid artery (LCCA), the left subclavian artery (LSA), and one of the common femoral arteries are surgically exposed. For precautions reasons, percutaneously, a guide wire is inserted into the contralateral femoral vein to enable to promote CPB via peripheral vessels if needed.

**Fig. 1:**
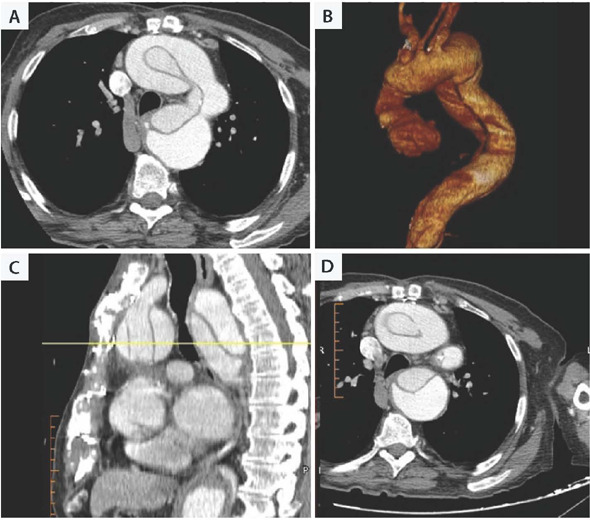
Preoperative computed tomography scan with reconstruction of the thoracic aorta showing the contact between the aneurismatic ascending aorta and the sternum.

**Fig. 2:**
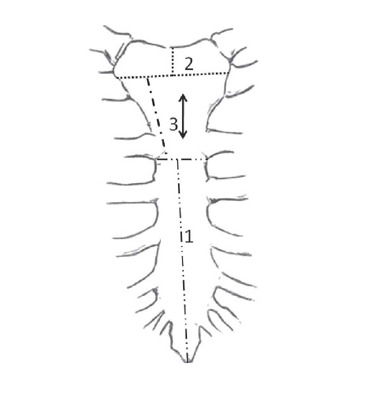
Two-stage sternotomy where partial sternotomy (1) is performed before heparin is given, and in phase two, a midline cut of the proximal segment (2) of the manubrium and paramedian cut (3) are done while the patient is connected to the extracorporeal circulation.

**Fig. 3:**
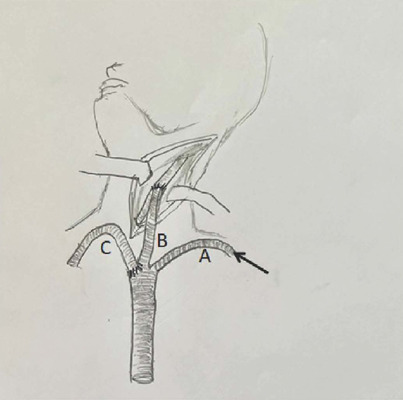
After heparin is given, a custom three-branch Dacron graft is anastomosed to the left common carotid artery (B). The lateral branch (A) is first used to be connected to the cardiopulmonary bypass to enable antegrade cerebral perfusion and, at last, to be anastomosed end-to-side to the left subclavian artery. Branch C is used for end-to-end anastomosis with the brachiocephalic artery.

**Fig. 4:**
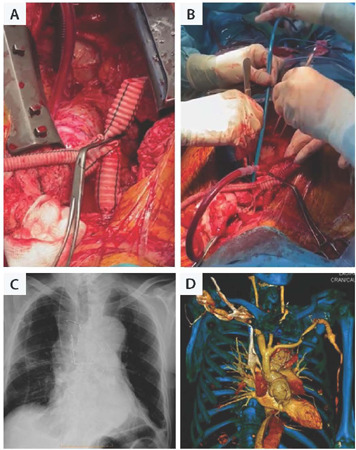
Intraoperative images of the antegrade cerebral perfusion via the custom-made graft (A) and the deployment of the thoracic endovascular aortic repair in antegrade fashion during circulatory arrest (B). Postoperative chest X-ray showing partial right hemisternum reinforcement (C) and computed tomography scan showing thoracic and epiaortic vessels reconstruction (D).

Following this, after having conducted a midline skin incision and removal of stainless-steel wires, with an oscillating saw, based on the computed tomography (CT) scan (Figure 1) showing the vessel to bone adhesion area, we perform the first stage partial longitudinal midline sternotomy from the third intercostal space down to the xiphoid process. A transverse T-shaped incision is performed in each hemi-sternum, sparing the manubrium (Figure 2). During the opening, the hemi-sterna are lifted vertically using Backhaus clamps which are placed temporarily on either side of the sternum, and systemic blood pressure is controlled pharmaceutically. Adhesions are dissected, allowing access to the heart and proximal ascending aorta. After heparin administration, the LCCA is clamped, and the stump pressure is verified. Using a custom-made three-branch graft (14 mm graft is anastomosed to the body of 14 × 7 × 7 mm), an end-to-side anastomosis is performed to the middle 7 mm branch graft (Figure 3). The patient is then connected to the CPB with the arterial return via the left femoral artery and venous drainage via two stage cannula which is placed in the right atrium. While cooling the body temperature to 30°C, the second stage of the sternotomy is completed, consisting of different cuts giving the possibility to "tackle" the adhesions from different sides, to expose the arch and vessels. During this stage, to reduce the risk of rapture, the CPB flow is reduced, thus lowering the blood pressure and the tension of the aortic wall. Once the aorta and epiaortic vessels are completely exposed, the brachiocephalic trunk is clamped, excised, and end-to-end anastomosed to the custom 14 mm side branch graft. The third lateral 7 mm branch graft is then connected to a separate CPB pump (the proximal three-furcated Dacron graft is clamped), an antegrade cerebral perfusion is instituted with a 10 ml/kg/min flow rate, and the LCCA is tied proximally (Figure 4A).

Cardiac arrest is achieved by blood cardioplegia, which is delivered to the aortic root if aortic cross-clamp can be positioned. Otherwise, in circulatory arrest, cardioplegia is delivered directly into the coronary ostia. In any case, once systemic circulation is interrupted, the “old” Dacron prosthesis is partially incised at its distal end, leaving a margin of about 2 cm of the prosthesis. Under direct vision, either hybrid graft or TEVAR, over a stiff guide wire, is inserted into the descending aorta, deployed, and sutured with running 3-0 polypropylene suture to the old Dacron graft, in order to guarantee a suture line sealing (Figure 4B). A longitudinal incision in the old Dacron prosthesis is then performed to create an orifice, and the branched graft, in end-to-side fashion, is anastomosed using 4-0 polypropylene suture. After de-airing, systemic perfusion is initiated, and the antegrade perfusion is interrupted. While the body is rewarmed, the third branched graft, which was previously used for the anterograde perfusion, is tunneled and anastomosed end-to-side to LSA (Figure 4D). At the end of surgery, partial hemisternum reinforcement is done do guaranty wound healing (Figure 4C)

### Clinical Experience

Based on preoperative CT scan, showing the aortic aneurysm close or with direct contact with the sternum, 10 consecutive patients (seven male, mean age 67 ± 5 years) underwent redo aortic arch replacement with debranching of the epiaortic vessels using either hybrid stent graft combined with Dacron graft or TEVAR. Time from the first procedure to the redo surgery was 62 ± 37 months (range four to 96 months). Extracorporeal circulation (ECC), cardiac arrest, and antegrade cerebral perfusion times were 157 ± 85, 64 ± 20, and 42 ± 18 min, respectively. Mean postoperative intubation time and intensive care unit stay were 9 ± 6 and 45 ± 26 hours. All patients experienced uneventful postoperative course and were discharged home

## DISCUSSION

In patients who have already undergone ascending aorta replacement, and particularly those who have experienced acute type A dissection, progression of the degenerative disease is expected. Thus, redoing the surgery to replace the aortic arch and descending aorta might be required. When the native aortic wall is adjacent to the sternum combined with severe adhesions, reentry to the mediastinum is technically challenging, characterized by long surgical time and high morbidity and mortality rates. Total endovascular approach has been proposed, however, to this day, it’s suggested only in selected cases and performed in highly specialized centers. In order to reduce surgical risks, epiaortic vessels debranching and extra-anatomic anastomoses using hybrid vascular grafts have been proposed^[5,6,7]^. However, when the aneurysm is close or adherent to the sternum, the risk of rupture during chest reentry is high, which results in catastrophic complications. Roselli et al.^[8]^ reported a high incidence of adverse events during reoperation, especially during dissection, and often when preoperative surgical risks were underestimated, resulting in poor patient outcome. To reduce such risks, alternative surgical and perfusion strategies should be considered^[9,10]^. Malvindi et al.^[1]^ reported that CPB was started before sternums reopening in over 30% of redo cases, including deep cooling and circulatory arrest in several cases. However, such strategy is characterized by prolonged CPB time, and dissection of adhesions, while the patient is heparinized, is incommodious, more complex, and time-consuming. Prolonged CBP time and circulatory arrest are known to be risk factors for neurologic adverse events, postoperative bleeding, and lung and kidney dysfunction, affecting negatively operative outcomes and hospital stay^[4]^. In our series, in comparison, ECC and cardiac arrest were notably shorter. Also, by guaranteeing continuous cerebral perfusion and short systemic arrest time, only moderate body temperature cooling is required.

In this contest, once the hybrid/TEVAR is deployed and sutured, systemic perfusion can be restarted resulting in short systemic ischemia time.

When redoing surgery to replace the aortic arch is planned, safety measurements and potential pitfalls should be taken into consideration. Based on careful evaluation of preoperative CT scan, balancing pro and cons, a surgical strategy should be planned and tailored to each patient. Accordingly, we exposed the relevant peripheral vessels and subsequently performed an end-to-side graft anastomosis to LCCA as part of the extra-atomic vessels debranching strategy enabling to promote selective cerebral perfusion in case of rapture of the aorta during adhesion dissection. To achieve optimal branches configuration, avoiding excessive graft length and risk of kinking at the end of the surgery, the side branch grafts length should be considered and measured prior to the distal anastomoses. An additional safety measure which we propose is the insertion of a guide wire into the femoral vein to enable percutaneous insertion of a femoral cannula and to promote CPB if needed. Moreover, external compression or occlusion through surgical exposure of the contralateral carotid artery should be considered in the preoperative surgical planning to avoid blood loss in case of rescue antegrade unilateral cerebral perfusion.

The two-stage sternotomy, avoiding in the first step the risky segment, allows access to the mediastinum, dissection of the adhesions before heparin administration, and central cannulation; in the second step, while on CPB, it enables completion of the sternotomy. As mentioned, based on the aorta-sternum reports, a paramedian sternotomy should be considered to further reduce the risk of damage to the weakened aortic wall. The sternum bone segmentation, which is conducted by different cuts, allows further adhesion dissection of the aorta attached to the manubrium from three directions, simplifying the procedure and avoiding vessel injury. If possible, leaving a small segment of the hemisterna attached to the clavicle will facilitate chest reconstruction at the end of the procedure, guaranteeing normal chest motion and uneventful wound healing.

Leaving a few centimeters of the old Dacron graft at the distal end represents several advantages when employing either TEVAR or hybrid stent grafts: first, performing the anastomosis is easier because both old and new Dacron grafts diameters are similar, and, differently from the native aortic wall, given the Dacron fabric resistance, anastomosis can be done faster.

In chronic dissection, when the ascending aorta has been previously replaced with a Dacron graft, we prefer the TEVAR on the hybrid graft because different diameters and lengths are available and the tapered version can be more appropriate to adapt to the diameter of the true lumen, hopefully reducing the risk of stent-induced new entry.

However, it should be emphasized that the proposed surgical strategy might represent some limitation. When rupture occurs during sternum opening, control of bleeding might be complex. In this circumstance, the heart and body perfusion might be compromised, resulting in intra and postoperative complications and high mortality rates^[8]^. Therefore, meticulous preoperative CT and surgical planning are mandatory. When pseudoaneurysm is detected, because of the absence of real aortic wall, the risk of rupture is higher, and reentry should be done in deep hypothermia and circulatory arrest. Also, the heart sternum contact should be carefully evaluated especially when in the right side (*i.e.*, right atrium is large and with thin wall).

Finally, multiple cuts in the sternum might complicate respiratory function and chest wound healing. Such adverse event can lead to postoperative respiratory disorders especially in patients with chronic obstructive pulmonary disease^[11]^.

## CONCLUSION

In conclusion, with careful preoperative planning guided by preoperative imaging, extra-anatomic epiaortic vessels reconstruction might simplify surgical procedure, reduce CPB, and improve patient’s outcomes.

## Abbreviations, Acronyms & Symbols

**CPB** =: **Cardiopulmonary bypass**
**CT** =: **Computed tomography**
**ECC** =: **Extracorporeal circulation**
**LCCA** =: **Left common carotid artery**
**LSA** =: **Left subclavian artery**
**TEVAR** =: **Thoracic endovascular aortic repair**

## References

[1] Malvindi PG, van Putte BP, Heijmen RH, Schepens MA, Morshuis WJ (2010). Reoperations for aortic false aneurysms after cardiac
surgery. Ann Thorac Surg.

[2] Bachet J, Pirotte M, Laborde F, Guilmet D (2007). Reoperation for giant false aneurysm of the thoracic aorta: how
to reenter the chest?. Ann Thorac Surg.

[3] Zeitani J, Buccisano F, Nardella S, Flaminio M, Prati P, Chiariello G (2013). Mini-extracorporeal circulation minimizes coagulation
abnormalities and ameliorates pulmonary outcome in coronary artery bypass
grafting surgery. Perfusion.

[4] Leone A, Beckmann E, Martens A, Di Marco L, Pantaleo A, Reggiani LB (2020). Total aortic arch replacement with frozen elephant trunk
technique: results from two European institutes. J Thorac Cardiovasc Surg.

[5] Ribeiro TS, Gadelha HP Júnior, Santos MAD (2021). Hybrid repair versus conventional open repair approaches for
aortic arch disease: a comprehensive review. Braz J Cardiovasc Surg.

[6] Zeitani J, Nardi P, Bellos K, De Propris S, Chiariello L (2013). Alternative surgical approach to treat aortic arch aneurysm after
ascending aortic replacement with hybrid prosthesis. Thorac Cardiovasc Surg.

[7] Akbulut M, Ak A, Arslan O, Dönmez AA, Taş S, Cekmecelioglu D (2020). Comparison between arch zones in modified frozen elephant trunk
procedure for complex thoracic aortic diseases. Braz J Cardiovasc Surg.

[8] Roselli EE, Pettersson GB, Blackstone EH, Brizzio ME, Houghtaling PL, Hauck R (2008). Adverse events during reoperative cardiac surgery: frequency,
characterization, and rescue. J Thorac Cardiovasc Surg.

[9] Schepens MA, Dossche KM, Morshuis WJ (1999). Reoperations on the ascending aorta and aortic root: pitfalls and
results in 134 patients. Ann Thorac Surg.

[10] Mohammadi S, Bonnet N, Leprince P, Kolsi M, Rama A, Pavie A (2005). Reoperation for false aneurysm of the ascending aorta after its
prosthetic replacement: surgical strategy. Ann Thorac Surg.

[11] Zeitani J, Pompeo E, Nardi P, Sergiacomi G, Scognamiglio M, Chiariello G (2013). Early and long-term results of pectoralis muscle flap
reconstruction versus sternal rewiring following failed sternal
closure. Eur J Cardiothorac Surg.

